# The unified rule of phyllotaxis explaining both spiral and non-spiral arrangements

**DOI:** 10.1098/rsif.2018.0850

**Published:** 2019-02-27

**Authors:** Takuya Okabe, Atsushi Ishida, Jin Yoshimura

**Affiliations:** 1 Graduate School of Integrated Science and Technology, Shizuoka University, 3-5-1 Johoku, Hamamatsu 432-8561, Japan; 2 Center for Ecological Research, Kyoto University, Otsu, Shiga 520-2113, Japan; 3 Department of Mathematical Systems Engineering, Graduate School of Science and Technology, Shizuoka University, Hamamatsu 432-8561, Japan; 4 Department of Environmental and Forest Biology, State University of New York College of Environmental Science and Forestry, Syracuse, NY 13210, USA; 5 Marine Biosystems Research Center, Chiba University, Uchiura, Kamogawa, Chiba 299-5502, Japan

**Keywords:** plant morphology, plant anatomy, convergent evolution, internal adaptation

## Abstract

Leaf-like appendages of different plant groups are arranged in common phyllotaxis patterns categorized into two types: spiral and non-spiral arrangements. The adaptive reason for this morphological convergence is unknown. In the non-spiral arrangement, the divergence angle between successive leaves is a simple fraction of 360°, e.g. distichy, decussate and whorled phyllotaxis. In the spiral arrangement, the divergence angle of nascent leaves at the shoot apex is fixed at the golden angle 137.5°, whereas those of the developed leaves varies within a sequence of Fibonacci fractions, such as 1/3, 2/5, 3/8, 5/13, etc. The optimality of the golden angle has been shown recently by assuming that the pattern of developed leaves varies during growth in a manner depending on the divergence angle of nascent leaves. Here we propose a unified rule of phyllotaxis to explain both types of arrangement: the developed leaves form vertical rows along the stem. In the non-spiral arrangement, nascent to developed leaves always follow this rule, so that the number of leaf rows is kept constant irrespective of stem growth. In the spiral arrangement, developed leaves attain this rule by adjusting the divergence angle from the golden angle. The spiral arrangement is adaptive in that the number of leaf rows varies during growth depending on shoot thickness.

## 1. Introduction

Leaf-like organs of most seed plants, ferns, mosses and even brown algae are arranged according to common rules, phyllotaxis [1–4]. Leaves of mosses (Bryophyta) and leaves of vascular plants are not the result of descent from a common ancestral structure. Brown algae is a lineage very distant from land plants [4–7]. Since the organization of meristems (growing tips) varies significantly among these groups, physiological mechanisms that lead to the same arrangement are probably different [8]. It is of great importance to understand what underlies this morphological convergence phenomenon. To the present day, phyllotaxis studies have been focused almost exclusively on morphogenesis [9]. In biology, however, it is as important to ask for ultimate causation to answer the ultimate ‘why’ question as to ask for proximate causation [10]. The ultimate or evolutionary factor for suppressing diversity in phyllotaxis is a fundamental open question. First and foremost, the rules of phyllotaxis apparently have nothing to do with environmental factors. Some pattern characterizes a group (distichy of grasses, Poaceae, decussate of the mint family, Lamiaceae, etc.), while closely related species may be distinguished by different pattern types (e.g. spiral versus whorled in *Sedum*). Generally, an ideal pattern is observed for a young, upright shoot before extrinsic factors to break radial symmetry, like gravity and sunlight, come into play. The arrangement of leaf insertions, phyllotaxis, should be distinguished from the orientations of laminae (flattened surfaces). The latter is vital for the functions of leaves while the former is not. In fact, the general laws of phyllotaxis are the rules on the angle of divergence between consecutive leaf insertions, divergence angle, even though the actual pattern of leaf arrangement depends on the other parameters significantly. It has been well recognized that the special arrangements of leaf surfaces are adjusted to external environments, such as light. For example, the leaves of plants growing under shaded understory arrange to escape the overlapping of each other leaves for increasing the light-capture efficiency [11]. By contrast, the leaves of plants growing under strong sunlight arrange to decrease the amount of absorbed excess light energy for reducing the risk of photoinhibition [12]. Thus, plants adjust leaf surfaces to their growing environments and this is achieved under the constraint of their own phyllotaxis rules. Another example is outwardly similar two-ranked arrangements of the bald cypress (*Taxodium distichum*) and the dawn redwood (*Metasequoia glyptostroboides*), which originate from different arrangements of spiral and decussate phyllotaxis, respectively. In phyllotaxis, not all theoretically possible patterns occur with comparable frequency. We do not yet know the reason why plants with different types of phyllotaxis are found in the same environment and why only selected types have been evolutionally maintained in most plant species.

**Figure 1.**
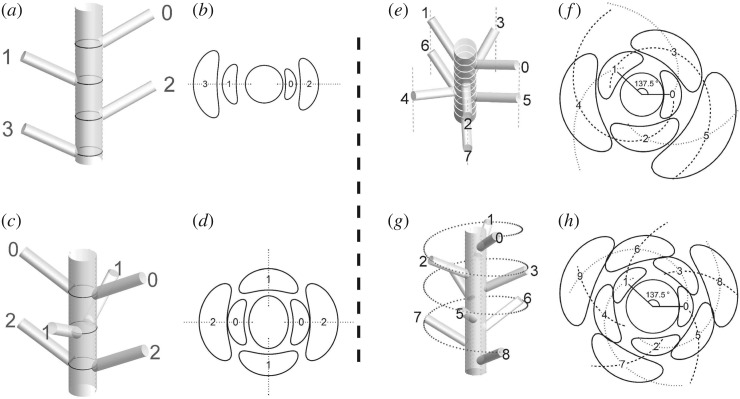
Two types of leaf arrangement: whorled (*a–d*) and spiral (*e–h*) phyllotaxis. (*a*) Distichy, or a 1/2 phyllotaxis. (*b*) Distichy at the shoot tip. Leaves are arranged in two rows (dotted). (*c*) Decussate phyllotaxis. (*d*) Decussate at the shoot tip, arranged in four rows (dotted). (*e*) 2/5 phyllotaxis of mature leaves. Five vertical rows are denoted by dotted lines. (*f*) 2∶3 phyllotaxis of nascent leaves. At the shoot tip, leaves form 2 (dashed) and 3 (dotted) curved rows as they are arranged with divergence angle 137.5°. (*g*) 3/8 phyllotaxis. The fundamental spiral is denoted by a dotted spiral. (*h*) 3∶5 phyllotaxis of nascent leaves, arranged with 137.5°.

Phyllotaxis patterns are generally classified into two categories, spiral and non-spiral arrangements [4,9,13]. Non-spiral arrangement consists of the alternation of a leaf or a whorl of leaves at each node. Distichy (figure 1*a,b*) and decussate (figure 1*c*,*d*) phyllotaxis are special cases of this type. The arrangement consists of vertical rows, called orthostichies (dotted lines in figure 1*b*,*d*). A distinctive characteristic of this arrangement is that the same pattern is preserved for nascent leaves (figure 1*b*,*d*) and mature leaves (figure 1*a*,*c*), the same because phyllotaxis focuses on divergence angle, the angle of rotation between consecutive leaves. In spiral phyllotaxis, however, nascent leaves at the shoot tip are arranged in curved spirals, called parastichies (figure 1*f*,*h*), while leaves on an elongated stem are arranged in longitudinal rows (orthostichies) (figure 1*e*,*g*) [13–15]. Whether spiral or non-spiral, the row pattern of mature leaves has a fractional value of divergence angle. The most commonly observed is a 2/5 phyllotaxis in which leaves are arranged in five vertical rows consisting of cycles of five leaves making two turns (figure 1*e*) [1–3]. It is empirically established that observed values of phyllotaxis fraction form a systematic sequence,

1.1 1/2, 1/3, 2/5, 3/8, 5/13, 8/21, etc.,

called the main sequence of phyllotaxis [2,3,13]. It has also been evidenced that the patterns of nascent leaves leading to this sequence (figure 1*e*,*g*) have a universal value of divergence angle, i.e. the golden angle 137.5° (figure 1*f*,*h*) [13,16–19]. This angle, about 0.382 of 360°, is the limit value of the above sequence (1.1). At a rough estimate, the angle 137.5° of nascent leaves and the angles in (1.1), expressed in degrees, are approximately the same. However, their difference is of primary importance. The angle 137.5° at the shoot tip is ‘non-fractional’, i.e. not approximated by a simple fraction, so that nascent leaves are not arranged in straight rows. The straight arrangement according to the main sequence (1.1) is secondarily caused from this curved 137.5° arrangement while the shoot stem elongates [20]. Since nascent leaves are not aligned radially (figure 1*f*,*h*), vascular strands run obliquely when they are formed. Elongation of the stem sets up tensions in the strands and the oblique course is straightened to establish a vertical arrangement of mature leaves by accompanying torsion of the whole stem [13,14,20]. In their initiation, every 2nd, 3rd, 5th, 8th, etc., leaf is directed towards nearly the same direction to make 2, 3, 5, 8, etc., curved rows (figure 1*f*,*h*). Phyllotaxis fraction is determined by which of them are connected and straightened up eventually. While two consecutive leaves define a divergence angle, the fractional representation is useful only after inter-leaf connections are established.

The phyllotaxis fraction concept has been widely used in the morphology of adult plants [5,21,22]. In plant anatomy, the fractional expression is of practical significance as it describes the network structure of vascular connections [23–25]. The past studies have revealed a close connection between phyllotaxis and internal structure, which suggests that regular phyllotaxis contributes to establishing evolutionarily optimal architecture of a growing shoot. In spiral phyllotaxis, the optimality of the 137.5° angle has been shown previously [26]. This angle occurs even in the arrangement of tentacles in jellyfish [27]. These studies attach significance to observed variability of the final arrangement of an adult individual. On the other hand, it has been known for a long time that lateral rootlets on a root are arranged in longitudinal rows [28] and two-ranked arrangement are found on a shoot in all groups of plants [29]. These facts have not been regarded as of special importance so far, because the longitudinal arrangement obviously conforms to the anatomical architecture. In accord with this empirical rule, this study puts forward a general view that the two major types of phyllotaxis originate from a common rule, i.e. mature organs are arranged in rows along the axis. The present hypothesis is based on the premise of the descriptive method in plant morphology, established in the nineteenth century [30]. The phyllotaxis fraction concept assumes that mature leaves form straight rows, whose number being the denominator. The row arrangement and its variations are correlated with the network structure of vasculature [13,20,23–25,31]. In spiral phyllotaxis, the row number varies depending on the relative size of primordia and the shoot apex (plastochrone ratio) [32]. Thus, the divergence angle is modified by rearrangement of the vascular structure (Rektipetalität) [13]. We assume this variation as an empirical fact and focus on its biophysical aspect. Although substantial progress has been made in molecular dynamics of phyllotaxis pattern formation [33,34], specific details of the molecular mechanisms underlying this rearrangement are yet to be investigated. In theory, the assumption of straight row formation raises a serious problem of consistency with another important observation. In spiral phyllotaxis, initiated leaves are not arranged in straight rows, so that the fractional description is invalid for them. The problem is to reconcile apparently conflicting observations in different stages. It is not obvious at all why leaves to be arranged in straight rows are initiated in curved rows. The present theory resolves this paradox as follows. The model assumes that leaves are initiated at constant intervals of angle and that a construction cost is incurred if the initiated leaves are not arranged in radial rows because they are arranged in rows on maturation. The lower the construction cost, the higher the fitness for the plant's survival. Evolution selects individuals to initiate leaves in a convenient manner for the architecture of the entire shoot. Non-spiral phyllotaxis is the simplest case of no cost, where the initiated leaves are arranged in the same row pattern as that of developed leaves. In this case, the leaf pattern is uniquely fixed from the outset. Spiral phyllotaxis is a non-trivial case of non-zero cost, where the initiated leaves are not arranged radially so that there are multiple ways of forming rows (e.g. 2/5 and 3/8 derive from 137.5°, figure 1*e*,*g*). In this case, the optimal arrangement of nascent leaves becomes uniquely non-radial, so to speak, as an average of the multiple row patterns to which it leads (the average of 2/5 and 3/8 is about the same as 137.5°).

## 2. Model

At the shoot tip, leaves are initiated at constant intervals of divergence angle *α*. As they develop, each leaf (numbered *n*) tends to form a row with an older leaf lying near to it (*n* + *m*) (e.g. *m* = 5 in figure 1*e*,*f* and *m* = 8 in figure 1*g*,*h*). This tendency of row formation exerts selective pressure for evolving the innate angle *α*. To take into account that the age difference *m* may vary in the course of growth, we assume that the lower bound *M* allowed for *m* (*M* ≤ *m*) varies with a relative frequency *w_M_* ($\sum_{M}w_{M}=1$). For *w*_2_ = 1 (*w*_3_ = 1), every second (third) leaves tend to stand in a row to result in a 1/2 (1/3) phyllotaxis. When *w*_4_ = 1, leaf *n* may be linked to leaves *n* + 4 and *n* + 5, but not to *n* + 2 and *n* + 3 as they are too close to *n* (i.e. *m* < 4). Since a row pattern has a common fraction value *α*_PF_ = *n*/*m* of divergence angle, the assumed tendency of row formation is expressed in terms of a fitness function peaked at a fraction *α*_PF_ near in value to *α*, i.e. *q*[*α*; *α*_PF_] = −(*α* − *α*_PF_)^2^. Weighted with the relative frequency (*w_M_*), mean fitness is given by

2.1 $$f=\underset{M}{\sum }w_{M}q[\alpha ;\alpha_{\mathrm{PF}}].$$

(For details, see electronic supplementary material.)

According to the convention, the angle *α* denotes the ratio to the full circumference, i.e. the angle is 360*α* in degrees. Measured in the spiral direction, the angle may take any value from 0 to 1/2 (180°) theoretically. Empirically, however, the observed values do not form a continuous spectrum. The empirical rules are that the angle is effectively fixed at 137.5° in most cases (normal spiral phyllotaxis) and 180° in some cases (distichy), while a few other constant angles like 99.5° (anomalous phyllotaxis) and 120° (tristichy) may occur though much less frequently [16–18]. For simplicity, higher-order patterns with more than five rows are neglected by assuming *w_M_* = 0 for *M* > 5.

## 3. Results

### 3.1. Constant phyllotaxis

**Figure 2.**
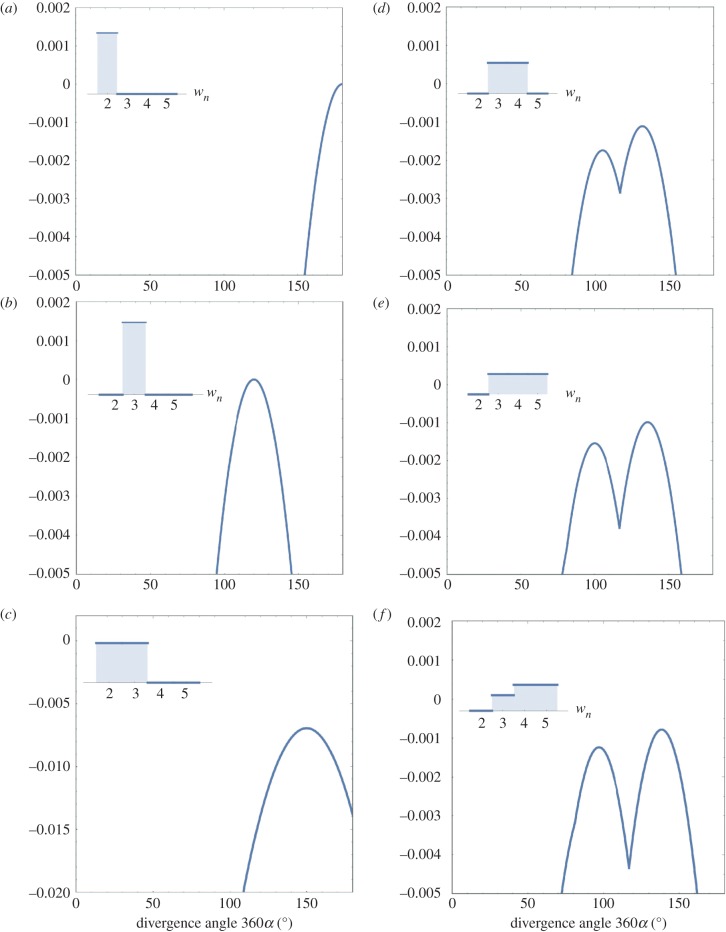
Fitness as a function of divergence angle *α* of nascent leaves. (*a*) Constant phyllotaxis (*w*_2_ = 1) with a peak at 180°. (*b*) Constant phyllotaxis (*w*_3_ = 1) with a peak at 120°. (*c*) Variable phyllotaxis (*w*_2_ = *w*_3_ = 1/2) with a peak at 150°. (*d*) Variable phyllotaxis (*w*_3_ = *w*_4_ = 1/2) with two peaks near 100° and 137.5°. (*e*) Variable phyllotaxis (*w*_3_ = *w*_4_ = *w*_5_ = 1/3). (*f*) Variable phyllotaxis (*w*_3_ = 0.23, *w*_4_ = *w*_5_ = 0.39). (Online version in colour.)

This is the case *w_M_* = 1 for a certain integer *M* ($w_{M^{\prime }}=0$ for *M*′ ≠ *M*). Since fitness *f* consists of a single term, optimal angle *α* is equal to a fractional value *α*_PF_. The simplest arrangement is a two-ranked pattern with *α*_PF_ = 1/2 for *w*_2_ = 1, i.e. *f* = −(*α* − 1/2)^2^ (figure 2*a*). This result is immediately generalized to alternating whorls of *N* leaves, *f* = −(*α* − 1/(2 *N*))^2^, where 2 *N* is the number of vertical rows. This general expression applies to distichy (*N* = 1; figure 1*a*,*b*) and decussate (*N* = 2; figure 1*c*,*d*). The next simplest is a three-ranked arrangement with *α*_PF_ = 1/3 (120°) for *w*_3_ = 1 (figure 2*b*).

### 3.2. Variable phyllotaxis

In variable phyllotaxis, *w_M_* < 1, optimal angle *α* becomes a mean of multiple fractional values. For *w*_2_ = *w*_3_ = 1/2, it is in the middle of 1/2 (180°) and 1/3 (120°), namely 150° (figure 2*c*). For *w*_3_ = *w*_4_ = 1/2, fitness *f* is peaked at 132° and 105° (figure 2*d*). Similarly, two peaks occur at 136° and 100° for *w*_3_ = *w*_4_ = *w*_5_ = 1/3 (figure 2*e*). The second peak is generally lower than the first peak, whereas the two peaks become the same height in special cases *w*_2_ = *w*_3_ = 0 and *w*_4_ + *w*_5_ = 1. This result accords with the empirical rule that a 1/4 phyllotaxis is far less common than a 2/5 phyllotaxis. For *w*_3_ = 0.23 and *w*_4_ = *w*_5_ = 0.39, two peaks are at 138° and 100° (figure 2*f*).

**Figure 3.**
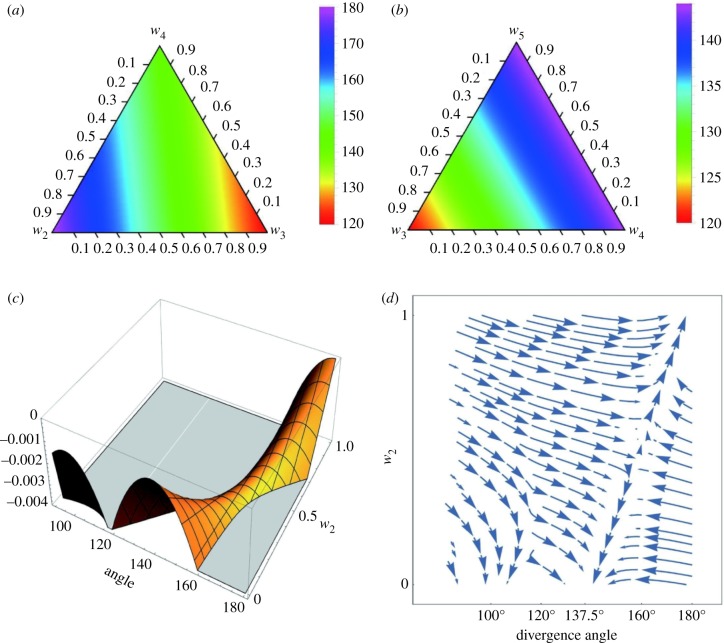
Optimal divergence angle and fitness landscape in variable phyllotaxis. Optimal angle is shown as triangle density plots for (*a*) *w*_2_ + *w*_3_ + *w*_4_ = 1 and (*b*) *w*_3_ + *w*_4_ + *w*_5_ = 1. (*c*) Fitness *f* and (*d*) evolutionary trajectories are plotted in a two-dimensional space of *α* and *w*_2_ (*w*_3_ = 0.23(1 − *w*_2_), *w*_4_ = *w*_5_ = 0.39(1 − *w*_2_). (Online version in colour.)

For three variables, optimal angle *α* is shown as density plots for *w*_2_ + *w*_3_ + *w*_4_ = 1 (figure 3*a*) and *w*_3_ + *w*_4_ + *w*_5_ = 1 (figure 3*b*). Each vertex of a triangle is a constant phyllotaxis, while the region inside the triangle is variable phyllotaxis (*w_M_* < 1). The right edge *w*_3_ = 0 in figure 3*b* is exceptional in that an anomalous angle 90° for a 1/4 phyllotaxis is equally optimal. For fixed ratios *w*_3_∶*w*_4_∶*w*_5_ = 0.23∶0.39∶0.39 (figure 2*f*), fitness *f* is shown as a three-dimensional plot on the optimal angle *α* and *w*_2_ (figure 3*c*). This plot has two peaks at (360*α*, *w*_2_) = (180, 1) and (138, 0), which correspond to distichy and normal spiral phyllotaxis, respectively. This result accords with the absence of arbitrary intermediate patterns. The saddle structure of low fitness occurs for any fixed ratios of *w*_3_, *w*_4_ and *w*_5_ if any of them is not identically zero. Evolutionary trajectories are drawn in a fitness landscape along gradient vectors of the fitness *f* (figure 3*d*).

## 4. Discussion

The process of phyllotaxis pattern formation is obviously goal-directed, the goal being the maintenance of a few selected patterns despite various environmental factors. The phyllotaxis phenomenon is a remarkable case of convergent evolution [30], the independent evolution of similar features in species of different lineages, e.g. seed plants, ferns, mosses, algae. Since leaf row formation is a larger scale phenomenon than pattern formation of initiated organs, their mechanisms are considered independently from each other. In spiral phyllotaxis, it is important to distinguish the phyllotaxis of the shoot apex and that of the mature shoot [14]. The latter is described in terms of regular fractions (1/3, 2/5, 3/8, …), while the former is not. The fractional expression describes the manner in which leaves are interrelated, so that this method is not strictly valid before vascular architecture (inter-leaf connection) is established. The various patterns originate from apparently different patterns of the shoot apex, which are actually the same phyllotaxis with a unique value of divergence angle (137.5°). The apex patterns are discerned by the numbers of two intersecting sets of secondary spirals, contact parastichies, which are two adjacent members of the Fibonacci sequence, 1, 2, 3, 5, 8, 13, 21, … (e.g. 2 : 3 and 3 : 5 in figure 1*f*,*h*, respectively). The occurrence of Fibonacci numbers is a mathematical consequence of the constant angle 137.5°. Therefore, there is a mathematical relationship (if not causal) between the parastichy numbers and the size of the shoot apex (e.g. smaller in figure 1*f* than in figure 1*h*) [32]. The apex size varies most significantly during the transition to a reproductive phase. Thus, the mature phyllotaxis varies as the apex size varies. The optimality of the 137.5° angle is proved by using a one-to-many correspondence between the divergence angle of nascent leaves and that of mature leaves [26,35] (see electronic supplementary material). This variation in spiral phyllotaxis is contrasted with its absence in non-spiral phyllotaxis. While the general laws of phyllotaxis are the rules of divergence angle, the other geometrical parameters, like organ size and internode length, play roles in causing variation in spiral phyllotaxis. In 5/13 and 8/21 shoots of *Linum*, leaf traces extend through about 12 and 19 internodes, respectively [31]. Thus, the higher the phyllotaxis fraction, the more internodes leaf traces traverse [23].

Two- and three-ranked arrangements (distichy and tristichy) are found in all groups of plants, while spiral arrangement occurs in plants with advanced body plans [3–5,29]. According to the categorization of the last section, the fixed tristichy (1/3) arrangement should be treated as an exceptional case of constant phyllotaxis. Although the fixed pattern of tristichy is spiral, it should be distinguished from the spiral phyllotaxis deriving from the golden angle. Brown algae show a variable phyllotaxis (1/2, 1/3, 2/5) as reflected in the stem morphology [5]. This primitive form of variable phyllotaxis may be compared with that of cacti and succulent euphorbias in which vertical rows are caused by the formation of rib structure [36]. In vascular plants, leaf rows are formed by the network of vascular connections. In plants with ideal phyllotaxis patterns, the leaf arrangement is strongly correlated with the vascular system [24,25]. Contrastingly, unstable phyllotaxis, e.g. in lycopods, is related to the irregularity of vascular system structure [8]. This observation is consistent with the present view that the ultimate tendency of leaf row formation evolves regular patterning mechanisms of phyllotaxis.

This study made a causal link between the empirical facts of different stages of development, i.e. the constant angle at the shoot tip and the fixed or variable phyllotaxis of the mature shoot. In the present model, various practical factors are not included in order to discuss, e.g. which type of phyllotaxis is more advantageous. Bravais & Bravais classified phyllotaxis into curviserial and rectiserial types [16,37], to which variable and constant phyllotaxis of this study correspond nicely. The former exhibits Fibonacci-related sequential patterns that derive from the golden angle 137.5° or a few other related irrational (non-fractional) angles [16]. Contrastingly, the latter (rectiserial) patterns are diverse as they may consist of any number of rows. Thus, it comprises any specific patterns of no general rule. Given the former's preponderance in nature, variable (curviserial) phyllotaxis should have more adaptive value than constant (rectiserial) phyllotaxis. Indeed, the former is advantageous in that the phyllotaxis expression (leaf row number, etc.) is flexibly changed as the size of the apical meristem, or shoot thickness, varies. Such ontogenetic changes are commonplace in a juvenile phase, and particularly conspicuous during the transition to a reproductive phase of seed plants. To take an instance, four-ranked decussate phyllotaxis of the dawn redwood (*Metasequoia glyptostroboides*) appears to place a constraint on the even distribution of branchlets and on the size of cones, as compared to spiral phyllotaxis, the most typical in confers.

In general, variability in phyllotaxis is not restricted within spiral phyllotaxis. Plant species with layered meristems show much more diverse phyllotaxis than those with segmented meristems and a single apical cell [29]. In many plants, phyllotaxis type changes at the shoot apex during ontogeny. In fact, dicotyledons and monocotyledons begin with decussate and distichy phyllotaxis, respectively, before spiral phyllotaxis is established. In conifer trees, different pattern types occur with different frequencies on the main stem and the lateral branch [38]. Thus, the variability of phyllotaxis is generally one aspect of phenotypic plasticity. Variable phyllotaxis discussed in this study is the structural consequence of variation in the size of the shoot apex. The conspicuous changes of phyllotaxis type are an aspect of heteroblasty, abrupt instead of a gradual change in the morphology of plants [39,40]. While a spiral phyllotaxis mutant with an atypical divergence angle is not known, *abphyl1* mutants of maize become decussate from distichy of the wild type [41]. Recently, the most intensive research has been carried out on the model plant *Arabidopsis thaliana*, for which a variety of phyllotaxis mutants are documented [33,34,42]. Since old times, various theoretical models have been put forward for the morphogenesis, or proximate mechanisms, of phyllotaxis pattern formation from physical, chemical, physiological and developmental standpoints [9]. However, the previous models of such approaches do not provide any clue to the basic problem of canalization, i.e. biological robustness. In the first place, they do not explain why the divergence angle is maintained at a constant value, not to mention why the change in phyllotaxis occurs from one type to another in a distinct manner. The current model is the first attempt to explain the adaptive reason for the regularity of observed leaf patterns.

The genetic control of phyllotaxis remains mysterious. Mutations that do not alter but phyllotaxis are not known. Not only irregular or disrupted phyllotaxis but conversion from spiral (alternate) to decussate (opposite) phyllotaxis may be caused as a secondary consequence of variation in the size of the shoot apical meristem. This study explained the adaptive significance of selected patterns, i.e. reduced variation (increased robustness). Although proximate cues of phyllotaxis changes are not known at all, this study may provide the groundwork for unravelling their underlying mechanisms. Thus, recent molecular approaches may be useful in detecting the proximate mechanisms controlling phyllotaxis variations.

Even though not all biological phenomena have an adaptive meaning, it is very implausible that such a designed property as the constant angle in phyllotaxis has none of it. While robustness in biological systems is different from robustness in engineered systems, design in the living system is likened to engineering design. The arrangement of *n* rows is compared to a tower building with *n* elevators. Depending on the floor area, there is an optimal row number. This is the problem of choice among vertical (non-spiral) arrangements. Thus, the common occurrence of small numbers (2 and 3) in different lineages of plants may not have any deeper meaning than they are just simple numbers. A further problem arises if the floor area varies depending on elevation. In this second problem, however, the analogy does not work because the constraints are specifically different in biology and design engineering. The golden angle and Fibonacci numbers in plants owe to the constraint that leaves are made in a spiral manner. The unique and ubiquitous solution would not be optimal if the constraint is relaxed in an arbitrary manner.

This study elucidated the ultimate or evolutionary factor for suppressing diversity in phyllotaxis in terms of a link between phyllotaxis and vasculature. We predict a positive correlation between the standard deviation (s.d.) in divergence angle of developing primordia and the s.d. in the angular difference between two developed leaves in a row (i.e. $\Delta \vartheta =\vartheta_{n+q}-\vartheta_{n}$ if the leaves are in a *p*/*q* phyllotaxis). This link should be directly verified by using empirical data in the future.

## Supplementary Material

Supplemental Materials

## Data accessibility

This article has no additional data.
