# Supplementary material for: The unified rule of phyllotaxis explaining both spiral and non-spiral arrangements
Source: J R Soc Interface. 2019 Feb 27;16(151):20180850. doi: 10.1098/rsif.2018.0850 (PMC6408360; doi:10.1098/rsif.2018.0850)
Supplement: Supplemental Materials [file rsif20180850supp1.pdf]

The unified rule of phyllotaxis explaining both spiral and non-spiral arrangements

Supplemental Materials

Takuya Okabe[1,] Atsushi Ishida[2] and Jin Yoshimura[3,4,5]

[1]Graduate School of Integrated Science and Technology, Shizuoka University, 3-5-1 Johoku, Hamamatsu 432-8561, Japan

[2]Center for Ecological Research, Kyoto University, Otsu, Shiga 520-2113, Japan.

[3]Graduate School of Science and Technology, and Department of Mathematical Systems Engineering, Shizuoka University, Hamamatsu 432-8561, Japan

[4] Department of Environmental and Forest Biology, State University of New York College of Environmental Science and Forestry, Syracuse, NY13210 USA.

[5] Marine Biosystems Research Center, Chiba University, Uchiura, Kamogawa, Chiba 299-5502, Japan.

Corresponding author: Takuya Okabe (okabe.takuya@shizuoka.ac.jp)

Supplementary Text

According to observations, the main sequence patterns of an elongated shoot (1/3, 2/5, 3/8, etc.) originate from the 137.5° arrangement at the shoot tip, depending on the vascular connections (fig. S1). The present model considers what longitudinal arrangements originate from a general angle different from 137.5°.

Three patterns for 360α=78.0, 99.5, and 137.5 degrees are shown in fig. S2*a*-*c*, where each arrow indicates the tendency to the nearest vertical arrangement. The first two angles

(78.0 and 99.5°) are of anomalous spiral phyllotaxis, which are rarely found in place of the 137.5° angle of normal spiral phyllotaxis. When α is close to these angles, fitness f is given by

$$f_a=-w_2(\alpha-1/2)^2-w_3(\alpha-1/3)^2-w_4(\alpha-1/4)-w_5(\alpha-1/5)^2,$$

$$f_b=-w_2(\alpha-1/2)^2-w_3(\alpha-1/3)^2-w_4(\alpha-1/4)-w_5(\alpha-1/4)^2,$$

and

$$f_c=-w_2(\alpha-1/2)^2-w_3(\alpha-1/3)^2-w_4(\alpha-2/5)^2-w_5(\alpha-2/5)^2,$$

respectively. Frequencies w_i (i=2~5) are non-negative parameters satisfying $w_2+w_3+w_4+w_5=1$. Since higher-order patterns are neglected for simplicity, no other case need be considered.

For $w_2=w_3=w_4=w_5=1/4$, the three functions are plotted in fig. S2*d*. Fitness f for general values of α and w_m is given by the maximum (most fitted) among the three cases,

$$f=\max[f_a,f_b,f_c].$$

To put it explicitly, $f=f_a$ for $0<\alpha<0.225$, $f=f_b$ for $0.225<\alpha<0.325$, and $f=f_c$ for $0.325<\alpha<1/2$. Two peaks in Fig. 2*d*-*f* are due to f_b and f_c. In general, f_c is larger than f_a and f_b, while f_b can be as large as f_c if $w_2=w_3=0$, $w_4+w_5=1$.

The peak of f_c lies at

$$\alpha=w_2/2+w_3/3+2(w_4+w_5)/5,$$

where $\partial f_c/\partial\alpha=0$. Thus, this optimal angle α is an weighted average of Schimper-Braun fractions 1/2, 1/3 and 2/5, which occur with relative frequencies w_2, w_3, and w_4+w_5,

respectively.

In principle, it is possible to evaluate the parameters w_i from the frequencies of occurrence of various patterns. In practice, however, no data has been published except in the original work of Bonnet [1]. Figure S3 shows irregular variations of the cycle 5 of a 2/5 phyllotaxis observed on branches of various plants. According to the top of the the fourth column, three 3-cycles and six 5-cycles were observed on a peach tree, indicating that 1/3 and 2/5 occur with a relative frequency of three 3's to six 5's, or w_3:w_4+w_5=9:30. Therefore, w_3=3/130.23 and w_4=w_5=5/130.39. These are used as representative values in Fig. 2*f* and Fig. 3*d* of the main text. The present model is so skeletal that high accuracy is not claimed for the 137.5° angle value. If the model is properly generalized to take account of higher-order patterns (like 3/8 and 5/13), optimal angle approaches to the ideal value 137.5° [2].

To visualize evolutionary trajectories, a further parameter has to be introduced. In terms of the ratio *c* of evolutionary rates of α and w_2, (α, w_2) evolves in the direction of $(\partial f/\partial \alpha, c\,\partial f/\partial w_2)$. In Fig. 3*b*, *c*=10.

1. Bonnet, C. 1754 *Recherches sur l'usage des feuilles dans les plantes*. Elie Luzac, fils.
2. Okabe, T. 2015 Biophysical optimality of the golden angle in phyllotaxis. *Scientific Reports* **5**, 15358.

Supplementary Figures S1-3

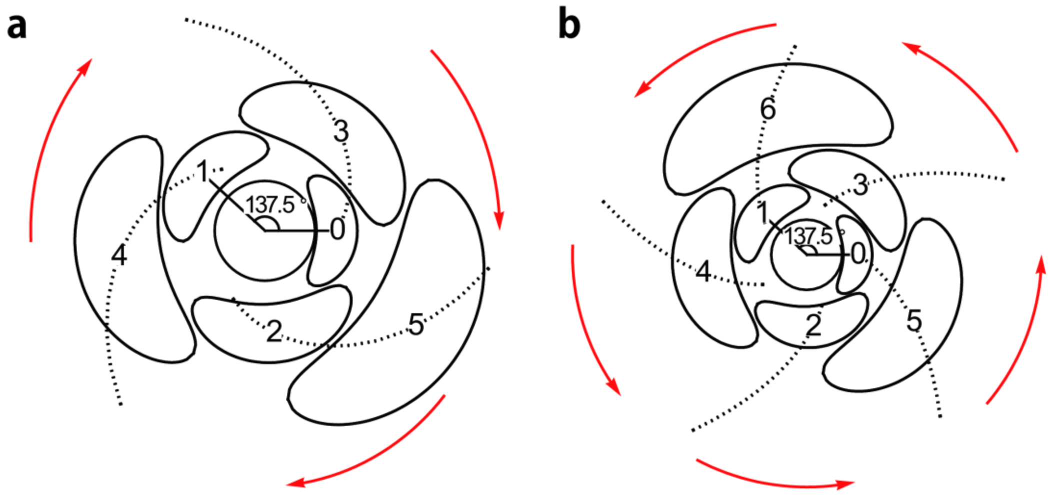

Figure S1: The 137.5° arrangement at the shoot tip gives rise to (*a*) 1/3 and (*b*) 2/5 phyllotaxis as the vascular bundles (dotted curves) are strengthened whereby the stem is twisted (arrows).

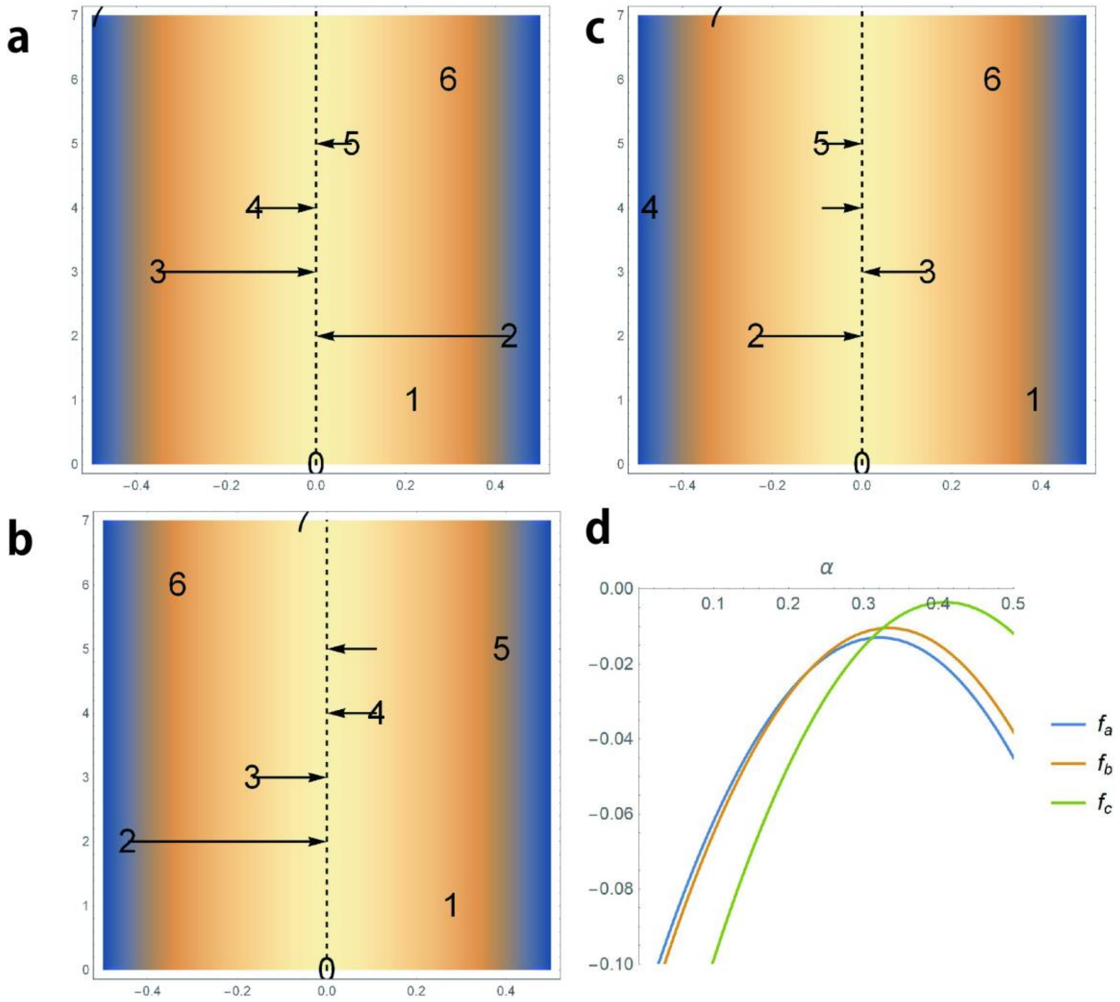

Figure S2: An arrow indicates tendency of leaves to a longitudinal arrangement (*a*) 360α=78.0 (α=0.216). (*b*) 360α=99.5 (α=0.276). (*c*) 360α=137.5 (α=0.382). (*d*) f_a, f_b and f_c as a function of α for w_2=w_3=w_4=w_5=1/4.

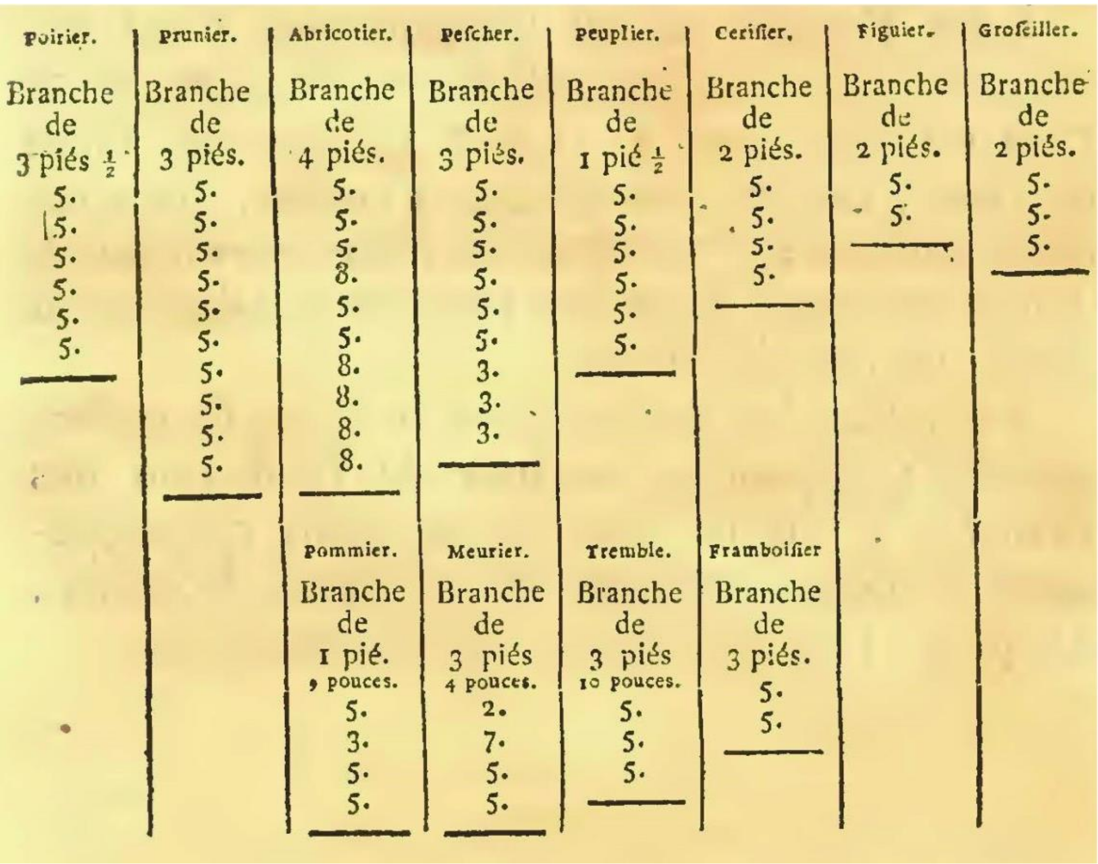

Poirier.	Prunier.	Abricotier.	Peſcher.	Peuplier.	Ceriſier.	Figuier.	Groſeiller.
Branche de 3 piés ½	Branche de 3 piés.	Branche de 4 piés.	Branche de 3 piés.	Branche de 1 pié ½	Branche de 2 piés.	Branche de 2 piés.	Branche de 2 piés.
5.	5.	5.	5.	5.	5.	5.	5.
5.	5.	5.	5.	5.	5.	5.	5.
5.	5.	5.	5.	5.	5.		5.
5.	5.	8.	5.	5.	5.		
5.	5.	5.	5.	5.			
5.	5.	5.	5.	5.			
	5.	8.	3.				
	5.	8.	3.				
	5.	8.	3.				
	5.	8.					

Pommier.	Meurier.	Tremble.	Framboiſier
Branche de 1 pié. 9 pouces.	Branche de 3 piés 4 pouces.	Branche de 3 piés 10 pouces.	Branche de 3 piés.
5.	2.	5.	5.
3.	7.	5.	5.
5.	5.	5.	
5.	5.		

Figure S3: Irregular variations of the cycle 5 of a 2/5 phyllotaxis on individual branches of various plants [1] (not in copyright).
